# Impact of Environmental Degradation on Human Health: An Assessment Using Multicriteria Decision Making

**DOI:** 10.3389/fpubh.2021.812743

**Published:** 2022-01-20

**Authors:** Ayesha Mumtaz, Erum Rehman, Shazia Rehman, Iftikhar Hussain

**Affiliations:** ^1^School of Public Administration, Hangzhou Normal University, Hangzhou, China; ^2^College of Public Administration, Zhejiang University, Hangzhou, China; ^3^School of Economics, Shandong University of Finance and Economics, Jinan, China; ^4^Department of Biomedical Sciences, Pak-Austria Fachhochschule, Institute of Applied Sciences and Technology, Haripur, Pakistan; ^5^Department of Mathematical Sciences, Karakoram International University Gilgit, Gilgit, Pakistan

**Keywords:** environmental pollution, public health, cardiac mortality, respiratory mortality, particulate matter, ground-level ozone, MCDA

## Abstract

Air pollution has emerged as a major global concern in recent decades as a result of rapid urbanization and industrialization, leading to a variety of adverse health outcomes. This research aims to investigate the influence of exposure to ambient and household particulate matter pollution (PM_2.5_), and ground-level ozone (O_3_) pollution on respiratory and cardiac mortality in Pakistan. We used grey incidence analysis (GIA) methodology to estimate the degree of proximity among selected variables and rank them based on mortality. Hurwicz's criterion is then adopted for further optimization by prioritizing the selected factors with the greatest influence on respiratory and cardiac mortality. The GIA findings revealed that asthma mortality is considerably impacted by exposure to ambient and household PM_2.5_ concentration while ischemic heart disease (IHD) mortality is potentially influenced by ground-level ozone exposure. Furthermore, results based on Hurwicz's analysis demonstrated that exposure to ambient PM_2.5_ concentration appeared as the most intensified factor of respiratory and cardiac mortality. This corroboration adds to the growing body of research demonstrating that exposure to ambient PM_2.5_ adversely leads to respiratory and cardiac risks, emphasizing the demand for further improvement of air quality in Pakistan. Besides, the suggested methodologies provide a valuable tool and additional practical knowledge for policymakers and decision-makers in drawing rational decisions.

## Introduction

Pakistan as a developing nation had the fifth-most polluted air in world in 2016, and positioned second in 2019 (1), owing to the same factors that plague much of South Asia: emanations from automobiles, industrial activities, such as block furnaces, industries, and power stations, and agricultural waste. This degree of air pollution is sabotaging well-being of Pakistanis, lowering down the median life expectancy by 2.7 years, comparative with what it would be if the WHO recommendation of 10 μg/m^3^ for long-term fine particulate matter (PM_2.5_) pollution was reached; and 2.2 years compared with the country's air quality benchmark of 15 μg/m^3^ (2). In 2016, 98% of population of Pakistan (nearly 200 million) resided in regions where yearly average PM_2.5_ pollution levels surpassed guidelines of the WHO. About 97% of the population inhabited regions where PM_2.5_ levels were higher than the threshold of country. PM_2.5_ concentrations have surged by 54% since 1998, leading to these high levels (3, 4).

The pathophysiologic effects of PM_2.5_ and O_3_ exposures in pulmonary systems have been widely studied, and it is evident that these particles can trigger and aggravate lungs infection in humans (5, 6). As the pulmonary and cardiac systems are complicatedly interconnected, it is conceivable that pulmonary oxidant stress characterized by PM_2.5_ and/or O_3_ exposure may cause downstream changes in the cardiovascular system. It is well-evidenced that particular environmental pollutants presented through the lungs can trigger and/or stimulate the development of cardiovascular disease (CVD) (7). However, many epidemiologic investigations have confirmed a strong link between PM_2.5_ and O_3_ and an increased risk of cardiac morbidity and mortality. In Pakistan, evidence comes from case studies revealing substantial variations in the outcomes, however, full-text original research articles are not more than a modest bunch (8–10).

**Figure 1:**
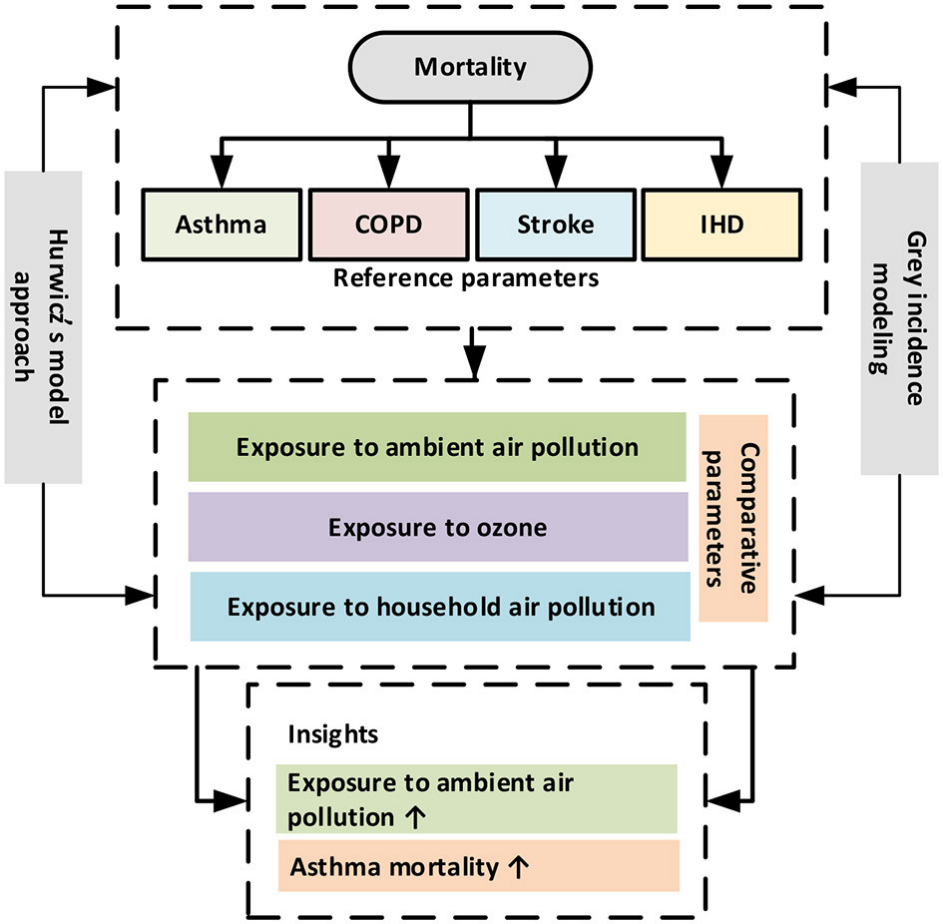
The visual abstract.

Given its significance, a wealth of research and analytic methodologies are utilized to explore the relationship between disease mortality and exposure to air pollutants across the world. In the Pakistani setting, there is a paucity of evidence relating to air pollutant exposure and the risk of mortality from chronic respiratory disease (CRD) and CVD. The lack of evidence has made it very difficult to assess the real situation in the Pakistani population. In response, the present study attempts to bridge the existing literature gaps by exploring the tie between exposure to ambient PM_2.5_ concentration, ground-level ozone (O_3_) exposure, household PM_2.5_ concentration exposure, and mortality from asthma, chronic obstructive pulmonary disease (COPD), stroke, and ischemic heart disease (IHD) in the Pakistani nation. However, by addressing exposure to PM_2.5_ (ambient and household) concentration and ground-level ozone with a wide range of disease mortality, we may provide a more dynamic spectrum of the interactions. To explore this association, we deployed a mathematical grey incidence analysis (GIA) modeling of grey system theory (GST) which comprised of Deng degree of GIA (DD-GIA), absolute degree of GIA (AD-GIA), and the second synthetic degree of GIA (SSD-GIA). The GIA models provide many advantages when contrasted with standard statistical models. For instance, they exhibit greater precision and may yield reliable outcomes with small sample size. Second, traditional statistical approaches, such as logistic regression, are inefficient for depicting the relations between variables in the biomedical domain, because of its dependency restrictions (11, 12). The GIA models may overcome this shortcoming as they are devoid of such assumptions. In addition, this study used Hurwicz's criterion to conduct a comparative analysis of all the selected factors and mortality (CRD and CVD) to ascertain which air pollutant is contributing more to mortality. The proposed methodologies are more appropriate when contrasted with other techniques for convincing outcomes and assist with avoiding endogeneity issues. The suggested model provides a valuable tool and additional practical knowledge for policymakers and decision-makers in drawing rational decisions to reduce air pollution and mortality in the Pakistani region. More importantly, this investigation may facilitate researchers with multiple criteria decision-making roadmap to help them enhance the quality of their studies and their understanding of how to use multiple criteria decision analysis (MCDA) techniques to evaluate and prioritize the influencing factors of disease mortality in environmental healthcare research. The visual abstract of the study is presented in Figure 1.

## Materials and Methods

### Data Source

For the period 2010–2019, information on the mortality (asthma, COPD, stroke, and IHD) were extracted from the Global Burden of Disease (GBD) study, harmonized by the Institute for Health Metrics and Evaluation (IHME), and is publicly accessible online (13). In addition, population-weighted exposure to ambient PM_2.5_ concentration (μg/m^3^), ground-level ozone (O_3_ in ppb) exposure, and household PM_2.5_ concentration exposure (μg/m^3^) abstracted from the State of Global Air report (2010–2019) (14). These parameters are estimated as the average exposure levels of population of country (urban and rural regions) to mean annual concentrations of PM_2.5_ and O_3_, separately. To evaluate PM_2.5_ exposures, the GBD scientist combines the number of people living in a specific region and the PM_2.5_ concentrations to which they are exposed. To evaluate ozone exposures, they combined the number of people living in a specific region and the surface ozone concentration to which they are exposed. This method determines human exposure to air pollutants in terms of the population-weighted average seasonal 8-h daily maximum concentrations for a specific region.

### Grey Incidence Decision Analyses

The data are analyzed by employing GIA methods (DD-GIA, AD-GIA, and SSD-GIA) (15, 16). These models are designed using SPSS (v26, IBM, NY, USA). Additionally, Hurwicz's methodology is employed to hierarchize the risk factors (air pollutants) that have a greater impact on CRD and CVD-related mortality within the Pakistani nation. The present analyses and modeling methods are deployed for the first time in the study to quantify the strength of influence and degree of correlation between selected factors and mortality (asthma, COPD, stroke, and IHD) in a multi-dimensional way. Deng's GIA model was employed to measure the partial closeness (a measure of influence) of the selected variables (ambient and household PM_**2.5**_ concentration exposure, ground-level ozone exposure, mortality from CVDs, and CRDs), whereas the bidirectional absolute GIA model was used to evaluate the integral closeness (degree of correlation) of the selected variables. The second synthetic GIA model was performed to obtain an overall estimate (weight) of inclusive proximity by accompanying the methods outlined in. The algorithms involved with the grey methods are explained in the following section.

#### Deng's GIA Model

Let *Y*_*i*_ = (*y*_*i*(1)_, *y*_*i*(2)_, ⋯  , *y*_*i*(*m*)_) be the basic/reference sequence addressing a dependent variable and *Y*_*j*_ = (*y*_*j*(1)_, *y*_*j*(2)_, ⋯  , *y*_*j*(*m*)_) be the arrangement of comparative sequences addressing independent variables, in the wake of going through initialing operator then, at that point grey relational gradient (GRG), the real number degree addressing the output of GIA model is depicted as γ_*ij*_ *or γ*(*Y*_*i*_, *Y*_*j*_) and can be accompanied by:


(1) $$\begin{array}{l} \gamma \left(Y_{i}\;,\;Y_{j}\right)=\;\frac{1}{m}\overset{m}{\underset{h=1}{\sum }}\gamma \left(y_{i\left(h\right)},y_{j\left(h\right)}\right) \end{array}$$

Where,


(2) $$\begin{array}{l} \gamma \left(y_{i\left(h\right)},y_{j\left(h\right)}\right)=\\ \frac{\min_{k}\min_{h}|y_{i\left(h\right)}-y_{j\left(h\right)}|+\zeta \;\max_{k}\max_{h}|y_{i\left(h\right)}-y_{j\left(h\right)}|}{\left|y_{i\left(h\right)}-y_{j\left(h\right)}|+\;\zeta \;\max_{k}\max_{h}|y_{i\left(h\right)}-y_{j\left(h\right)}\right|} \end{array}$$

Here, ζ* ϵ* (0, 1) represent as a distinguishing coefficient, and its value is generally considered to be ζ = 0.5. The implementation of the Deng's GIA model for evaluating the effect of one parameter/variable on the other has been highlighted in the literature (17, 18).

#### Bidirectional Absolute GIA Model

If *Y*_*i*_ = (*y*_*i*(1)_, *y*_*i*(2)_, ⋯   , *y*_*i*(*m*)_) *and Y*_*j*_ = (*y*_*j*(1)_, *y*_*j*(2)_, ⋯   , *y*_*j*(*m*)_) are two data sequences representing two variables associated with a system, then the algorithm to calculate the bidirectional absolute GRG is listed below.


(3) $$\begin{array}{l} \epsilon_{ij}=\frac{1+|r_{i}|+|r_{j}|}{1+|r_{i}|+|r_{j}|+|r_{i}-r_{j}|} \end{array}$$

Where,


(4) $$\begin{array}{l} r_{i}=\;\int_{1}^{m}Y_{i}^{0}dt,\;r_{j}=\int_{1}^{m}Y_{j}^{0}dt,\;r_{i}{-\;r}_{j}\\ =\;\int_{1}^{m}\left(Y_{i}^{0}{-\;Y}_{j}^{0}\right)\;dt \end{array}$$

(5) $$\begin{array}{l} Y_{i}^{0}=\left(y_{i\left(1\right)}^{0},\;y_{i\left(1\right)}^{0}\;,\cdots \;,\;y_{i\left(m\right)}^{0}\right) \end{array}$$

(6) $$\begin{array}{l} Y_{j}^{0}=\left(y_{j\left(1\right)}^{0},\;y_{j\left(1\right)}^{0}\;,\cdots \;,\;y_{j\left(m\right)}^{0}\right) \end{array}$$

(7) $$\begin{array}{l} Y_{i\left(h\right)}^{0}=\;y_{i\left(h\right)}-\;y_{i\left(1\right)}\;and\;Y_{i\left(h\right)}^{0}=\;y_{i\left(h\right)}-\;y_{i\left(1\right)}\\ h=1,2,\cdots \;,m \end{array}$$

Next, compute the bidirectional absolute GRG (ϵ^±^), provided by (19).


(8) $$\begin{array}{l} \epsilon^{\pm }=\{\begin{array}{c} +max\left(\epsilon_{ij},\epsilon_{ij}\right),=\epsilon_{ij}-\epsilon_{ij}>0\\ +\min\left(\epsilon_{ij},\epsilon_{ij}\right),=\epsilon_{ij}-\epsilon_{ij}<0 \end{array} \end{array}$$

Here, the –ve sign addresses an inverse grey relation (i.e., inversely proportional), while the +ve sign addresses a direct grey relation, and the magnitude to which either of these signs is appended indicates the intensities of the grey relation (i.e., degree of integral closeness/proximity).

#### Second Synthetic GIA Model

The SS-GIA model is an approach to estimate SS-GRG and can be acquired by utilizing the accompanying equation.

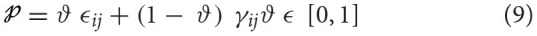



where ′
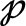
′ stands for the SS-GIA, ′ϵ′ for the absolute GIA, and ′γ′ for the Deng's GIA between the two grey datasets Y_i_ and Y_j_. When a decision-maker desires a holistic assessment that evenly integrates the benefits of both ′ϵ′and ′γ′ without preferring one over the other and may keep ϑ at 0.5. In the case of preferring is fundamental, then, at that point, the value of ′ϑ′ can be adjusted (20). If one desire to prefer ′γ′, then ′ϑ′ can be diminished, and assuming one desires to prefer ′ϵ,′ then ′ϑ′ can be increased. In the present investigation, we thought of ϑ = 0.5. Furthermore, in the SS-GIA equation, the absolute GIA was substituted by the bidirectional absolute GIA (without taking signs). When the interactions within the frameworks/systems are ambiguous, it is likewise advised that the absolute GIA can be supplanted by the bidirectional absolute GIA. Deng's GIA is established on grey incidence/correlation/relational coefficients of specific points, while absolute GIA is based on an integral (generally far-reaching) perspective, however, the SS-GIA is based on specific points and integral perspectives and shows overall proximity (correlation/closeness) (21, 22).

### Hurwicz's Criterion

The issue of multiple objectives often exists in the problems within systems, increasing the ambiguity of decisions. In this setting, to minimize errors, it is significant to find techniques that include the greatest number of criteria in the decision-making process that direct influence decisions. However, most of the time this technique is not easy to execute, because in many cases the decision-making criteria vary, raising the degree of uncertainty of the final response. In the literature, there are multiple decision-making (MCDM) techniques that facilitate researchers to make decisions and analyze preferred alternatives. These techniques are regarded for a variety of reasons. Researchers believed that the procedure allows visibility, consistency, and precision toward a more reasonable priority setting from a methodological standpoint. In a broader sense, the MCDA application procedure is regarded as an efficient and successful in priority settings. In this regard, the Hurwicz's criterion is one of the most recognized techniques in the literature which integrates a measure of both by giving optimism a specific percentage weight and pessimism the remainder (23). For each action choice, a weighted average with an alpha-weight, known as the coefficient of realism, could be determined. The decision-maker chooses the α subjectively. Determining a value for α concurrently results in a pessimistic coefficient of (1–α), which indicates the sensitivity of decision-makers to risk (23). For each action alternative B_i_ in B, a Hurwicz's weighted average ‘H’ may now be computed as follows:

H (B*i)* = α (row maximum) + (1–α) (row minimum)–for a maximization approach

H (B*i)* = α (row minimum) + (1–α) (row maximum)–for a minimization approach

Hurwicz's decision rule is used in the following steps.

1. Determine the value of the optimism coefficient α. Take note that 0 ≤ α ≤ 1.

2. Calculate the Hurwicz weighted average H for each action alternative.

Select the action alternative with the highest H as the final decision (maximum for a maximization problem, and minimum for a minimization problem).

## Results

**Figure 2:**
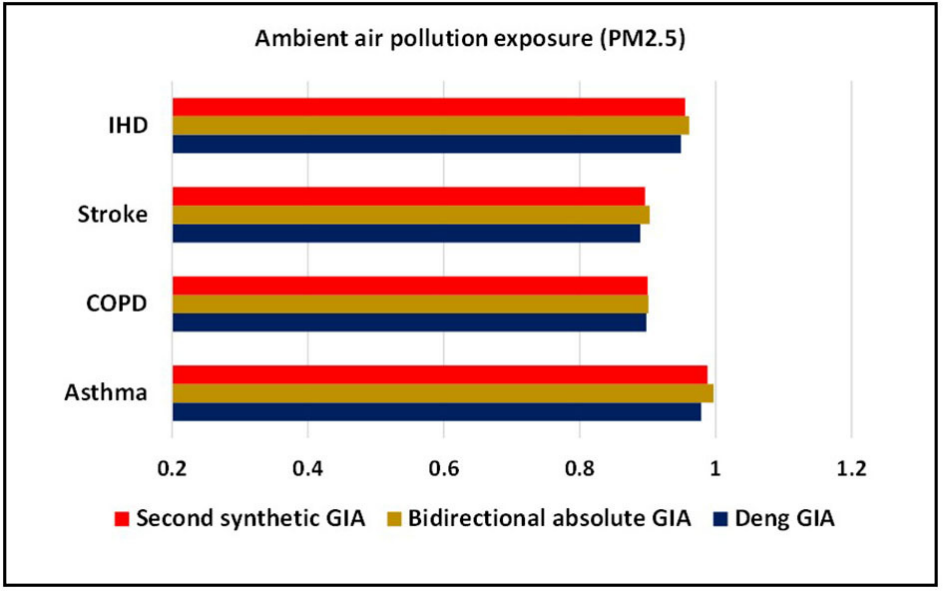
Comparative assessment of ambient air pollution exposure based on grey incidence analysis (GIA).

**Figure 3:**
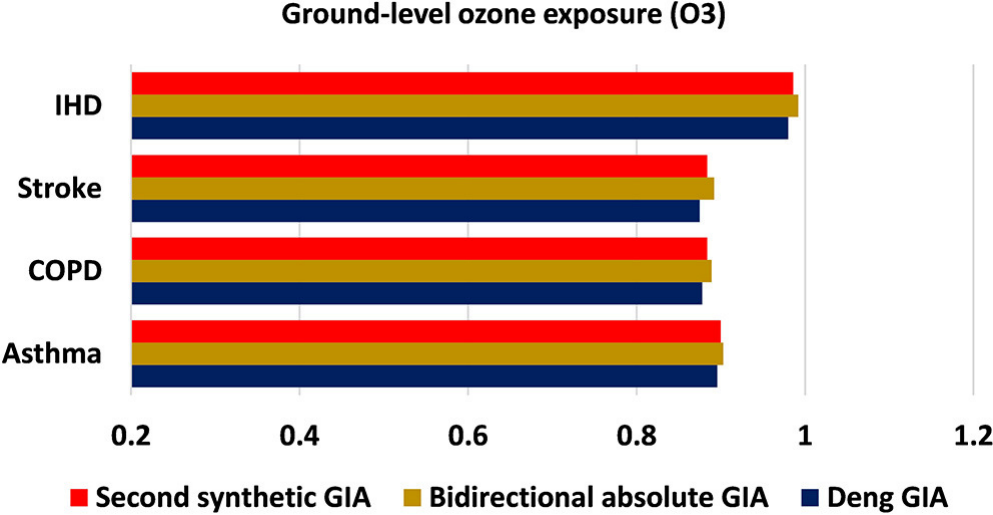
Comparative assessment of ground-level ozone exposure based on GIA.

**Figure 4:**
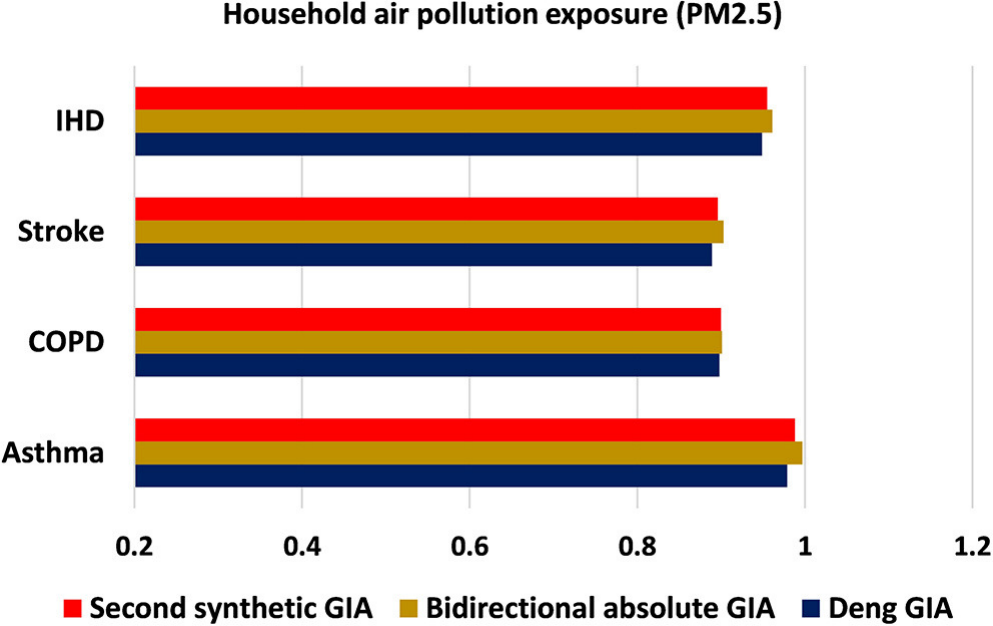
Comparative assessment of household air pollution exposure based on GIA.

The present investigation is carried out using grey approaches to quantify the strength of association between asthma, COPD, stroke, and IHD mortality with associated risk factors (exposure to ambient PM_**2.5**_ concentration, exposure to ground-level ozone, and exposure to household PM_**2.5**_ concentration) for the years 2010–2019. Table 1 demonstrates the outcomes of grey models, namely, the Deng's GIA, absolute GIA, and the SS-GIA for respiratory and cardiac mortality with associated risk factors. The absolute GIA and the SS-GIA models have values ranging from 0 to 1, whereas, Deng's GIA has values ranging from 0.5 to 1. It is also considered highly associated if it is near to 1 and weak if it diverges from 1. The graphical representation of the comparative assessment based on GIA can be seen in Figures 2–4.

**Table 1:** Grey incidence assessment between mortality and associated factors.

**Mortality**	**D-GRG (γ)**	**BA-GRG (ϵ^±^)**	**SS-GRG (** 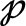 **)**	**Rank**
**Ambient air pollution exposure (PM**_**2.5**_ **)**				
Asthma	0.985	(0.994)	0.990	1st
COPD	0.870	(0.874)	0.872	4th
Stroke	0.890	(0.895)	0.893	3rd
IHD	0.979	(0.984)	0.982	2nd
**Ozone exposure (O**_**3**_ **)**				
Asthma	0.896	(0.903)	0.900	2nd
COPD	0.878	(0.889)	0.884	4th
Stroke	0.875	(0.892)	0.884	3rd
IHD	0.980	(0.992)	0.986	1st
**Household air pollution exposure (PM**_**2.5**_ **)**				
Asthma	0.979	(0.997)	0.988	1st
COPD	0.898	(0.901)	0.900	3rd
Stroke	0.889	(0.903)	0.896	4th
IHD	0.949	(0.961)	0.955	2nd

*D-GRG (γ), Deng grey relational gradient; BA-GRG (ϵ^±^), bidirectional absolute grey relational gradient; SS-GRG (p), second synthetic grey relational gradient; COPD, chronic obstructive pulmonary disease; IHD, ischemic heart disease. Inverse relationships are indicated by values enclosed in round parenthesis. SS-GRG is estimated without the use of signs. Response variable: mortality from asthma, COPD, stroke, and IHD*.

Ambient PM_2.5_ exposure is regarded among the most significant risk factors for CRD and CVD-related comorbidity and mortality, both of which are serious public health concerns (24). In the present analyses, as indicated by Deng's GIA model findings, a more grounded measure of influence between ambient PM_2.5_ exposure and asthma mortality in the Pakistani populace (0.985: first) is observed whereas, a most fragile level of influence is seen for COPD (0.870: fourth). The higher impact measure of asthma mortality portrays that the factors are unequivocally interconnected with one another. Then again, the same succession showed up from the findings of the bidirectional absolute GIA model. The degree of correlation is found much higher for mortality from asthma against ambient PM_2.5_ exposure with an estimated grey weight of 0.994. At a more aggregate level, as per SS-GIA findings, exposure to ambient PM_2.5_ is distinguished as the major contributor in accelerating mortality from asthma when compared with COPD, stroke, and IHD and ranked top. After asthma, IHD appeared to be more affected from exposure to ambient PM_2.5_ with an estimated weight of 0.982 and ranked second, trailed by stroke and COPD with an estimated degree of 0.893 and 0.872, respectively. When compared with COPD and CVD mortality, the greater degree of correlation between asthma mortality and ambient air pollution exposure portrays that those variables are significantly associated with each other (Table 1). These findings are aligned with the findings by Ilyas et al. (9) and Yamamoto et al. (25).

Exposure to the ground-level ozone potentially exacerbates a multitude of health complications, particularly respiratory and cardiovascular illnesses. Ozone levels are expected to rise in many regions of the world, resulting in an upsurge in ozone-related deaths and morbidities (26). Considering the impact of ground-level ozone exposure on mortality from CVD and CRD in Pakistan, asthma, COPD, stroke, and IHD all sustained their ranking order across all grey incidence models; however, the strength of influence and correlation was determined to be greater for IHD mortality. As per Deng's GIA estimates, mortality from IHD (0.980: first) tends to be more strongly influenced by ground-level ozone exposure when compared with asthma, COPD, and stroke, though the least fortunate influence is seen with stroke mortality (0.875: fourth). Nonetheless, as per bidirectional absolute GIA model findings, mortality from IHD (0.889: first) gave off an impression of being significantly associated with exposure to ozone concentration and situated top among the rest of the disease mortality. In general, mortality from stroke and COPD was shown to have the least association from ground-level ozone exposure by acquiring grey incidence (relational/association) weights of 0.884 (rank: third) and 0.884 (rank: fourth), respectively. Broadly speaking, exposure to ozone concentration had all the earmarks of being a potential risk factor in assessing and anticipating mortality from IHD in the population of Pakistan when compared with stroke and CRDs. These findings back-up prior investigations substantiating the adverse impact of ground-level ozone exposure in the development and progression of respiratory and cardiac disorders (27, 28).

Table 1 shows a review of the relationship between household PM_**2.5**_ exposure and mortality from CVD and CRD in the Pakistani populace using grey incidence models. Given the weights determined by Deng's GIA model findings, the highest influence measure is observed in the case of asthma mortality (0.979: rank: first) due to household PM_**2.5**_ exposure, whereas the most fragile measure is seen in the case of stroke (0.889: rank: fourth). The assessment based on bidirectional absolute GIA outcomes revealed the same sequence of ranking depicting asthma mortality (0.997: rank: first) is highly significant with exposure to household PM_**2.5**_ concentration. As a general view, based on the outcomes of the SS-GIA model, mortality from asthma and IHD is shown to be strongly associated with exposure to household PM_**2.5**_, whereas COPD and stroke are determined to be less associated. In general, exposure to household PM_**2.5**_ is determined to be potentially impacting asthma and IHD mortality in the Pakistani population with an estimated degree of association of 0.988 and 0.955, respectively. The scope of epidemiological investigations is steady with the findings of the current study depicting a strong association between household PM_**2.5**_ pollution and a variety of respiratory and cardiac disorders and associated mortality (25, 29, 30). The ranking order based on grey modeling analysis is as follows.

**Exposure to ambient PM**_**2.5**_ **concentration**

Asthma > IHD > Stroke > COPD

**Exposure to ground-level ozone (O**_**3**_ **) pollution**

IHD > Asthma > Stroke > COPD

**Exposure to household PM**_**2.5**_ **concentration**

Asthma > IHD > COPD > Stroke

To distinguish the potential risk factor in raising mortality from CRD (asthma, COPD) and CVD (stroke, IHD) in the Pakistani populace, Hurwicz's criterion was applied. Before applying it, one needs to create a decision scheme as shown in Table 2. Let, *p* = 4, *k* = 3, and outcome = *v* (*P*_*k*_, *D*_*p*_), whereas *k* = 1, 2, 3 and *p* = 1, 2, 3, 4. Let P_1_, P_2_, and P_3_ represent risk factors of mortality, and *D*_1_, *D*_2_, *D*_3_, and *D*_4_ represent mortality from asthma, COPD, stroke, and IHD, respectively. Table 3 represents the SS-GIA based matrix between the decision criteria (*D*_1_*-D*_4_) and decision actions (*P*_1_*-P*_3_).

**Table 2:** Defining the decision variables/parameters.

**Parameters**	**Measuring grey correlation between mortality (asthma, COPD, stroke, IHD) and associated risk factors**
Disease mortality (*D_*p*_*);	Asthma (*D_1_*), COPD (*D_2_*), Stroke (*D_3_*), IHD (*D_4_*)
*p* = 1,2,3,4	
Air pollution exposure (*P_*k*_*);	Ambient air pollution exposure (P_1_)
*k* = 1,2,3	Ozone exposure (P_2_)
	Household air pollution exposure (P_3_)

**Table 3:** Grey decision matrix.

	**D_**1**_**	**D_**2**_**	**D_**3**_**	**D_**4**_**
P_1_	0.999	0.872	0.893	0.982
P_2_	0.900	0.884	0.884	0.986
P_3_	0.988	0.900	0.896	0.955

### Hurwicz's Criterion Result

Among the chosen variables (*P*_1_*-P*_3_), to identify the potential risk factor of mortality (asthma, COPD, stroke, and IHD), Hurwicz's approach is employed. For the present investigation, we need to minimize the mortality, so the decision of Hurwicz's criterion would be characterized as follows by keeping alpha-weight at 0.3 for an optimistic approach, whereas 0.7 for a pessimistic approach.


$$\begin{array}{l} \min P_{k}\left\{\alpha \;\min\;D_{p}\;v\;\left(P_{k},D_{p}\right)+\left(1-\alpha \right)\;max\;D_{p}\;v\;\left(P_{k},D_{p}\right)\right\} \end{array}$$

The calculated weighted average by Hurwicz's criterion is presented in Table 4.

**Table 4:** Hurwicz's evaluations.

**Optimistic approach: by keeping** **α** **=** **0.3 and thus, 1–α** **=** **0.7**			
P_1_	(0.3 × 0.872) + (0.7 × 0.990)	0.9547	min
P_2_	(0.3 × 0.884) + (0.7 × 0.986)	0.9558	
P_3_	(0.3 × 0.896) + (0.7 × 0.988)	0.9613	
**Pessimistic approach: by keeping α = 0.7 and thus, 1–α = 0.3**			
P_1_	(0.7 × 0.872) + (0.3 × 0.990)	0.9074	min
P_2_	(0.7 × 0.884) + (0.3 × 0.986)	0.9146	
P_3_	(0.7 × 0.896) + (0.3 × 0.988)	0.9236	

Given Hurwicz's criterion outcomes for both optimistic and pessimistic approaches, exposure to ambient PM_**2.5**_ concentration gave off an impression of being a more intense risk factor in accelerating mortality (asthma, COPD, stroke, and IHD) among the selected risk factors. At a more aggregate level, the findings revealed that exposure to ambient PM_**2.5**_ concentration is a potential contributor to mortality from CVD (stroke and IHD) and CRD (asthma and COPD) in the populace of Pakistan. As per the WHO recommendations, air quality in Pakistan is unhealthy; the most current statistics show that the annual mean concentration of country of PM_**2.5**_ is 58 g/m^3^, which exceeds the recommended level of 10 g/m^3^. The most significant contributors to these air pollutants in metropolitan areas are inadequate energy consumption, a spike in the number of vehicles driven regularly, an increase in uncontrolled industrial emissions, and the combustion of waste and plastic (31, 32). Thus, for the synergistic reduction of air pollutants, a holistic management framework integrating health, energy, climate, and environment sectors should be designed.

## Discussion

The considerable loss of life and comorbidities synonymous with non-communicable diseases (NCDs) in developing countries, such as Pakistan, entails a rigorous assessment of all the relevant factors, from an individual up to the public level. Addressing this subject can make a significant impact on overall general public health improvement. Nonetheless, relatively few investigations have emphasized the assessment of air pollutant exposure and mortality from CVD and CRD in Pakistani settings *via* an MCDA approach. To the best of the authors' knowledge, the study is the first of its kind to measure the intensity and nature of the relationship between exposure to air pollutants specifically PM_**2.5**_ and O_**3**_, and mortality from asthma, COPD, stroke, and IHD in the Pakistani setting by employing grey modeling. Consideration of endogeneity concerns in the acquisition of mortality-related variables is one contribution to this type of study. MCDA methods could be beneficial in facilitating appropriate policy solutions. Such investigations could assist improve our capacity to acquire valuable insights into the multifaceted nature of the variables in a system.

Given the GIA findings, exposure to ambient and household PM_**2.5**_ concentration had all the earmarks of being profoundly associated with mortality from asthma, while ground-level ozone exposure seemed, by all accounts, to be exceptionally associated with IHD mortality among the chosen variables. In the outcomes of Hurwicz's analysis, exposure to ambient PM_**2.5**_ demonstrated to be a more heightened factor in influencing mortality from CRD and CVD among the chosen factors. To be more specific, our findings emphasize the importance of planned urbanization, sustainable population growth, energy-efficient local transportation frameworks, adaptation of clean and renewable energy sources, and city-wide tree planting to avoid further environmental and population health deterioration.

In light of the outcomes, this investigation suggested that ambient concentration of PM_**2.5**_ might be the key influencing factor of mortality from asthma followed by IHD, stroke, and COPD within the Pakistani nation. Pakistan has experienced swift urban growth since its foundation, and the pace has intensified in recent decades. Presently, ~50% populace of Pakistan lives in metropolitan regions. Individuals commute to metropolitan regions for better job prospects and significant ventures are arranged nearby urban areas. Furthermore, rural regions have inadequate medical and educational facilities, and land ownership among rural residents is diminishing. These are different components instigating rural residents to relocate to metropolitan regions (33, 34). In Pakistan, the industry has grown at an exponential rate; thus, urbanization degrades the environmental quality indirectly via industrialization. Furthermore, due to the poor public transit infrastructure in the urban regions of Pakistan, inhabitants opt for private transportation, which has led to massive automobiles emissions that contribute to environmental deterioration. Consequently, urbanization in Pakistan causes pollution and worsens population health over the long haul (35, 36).

Pakistan has insufficient energy sources. As per Demographic Health Survey, 62% of Pakistani households cook with biomass fuels (e.g., wood, coal, and agricultural, and animal wastes) (37). PM_**2.5**_ concentrations emitted by biomass fuel use have a significant impact on cardiac morbidity and mortality worldwide. Individually, the amount of fuel burned in a household might be significantly less than the amount used in industries. However, its influence on population health is far stronger because of its pervasive and continuous existence in the internal environment and the maximum time spent inside by humans (38, 39). This issue is quite possibly the most ignored area of the disease burden in the Pakistani region. It is indeed not hard to establish a tight connection between household PM_**2.5**_ concentrations exposure and health risks in humans. To minimize indoor air pollution during culinary activities, a variety of treatments are available. Changes in energy technology and boosting public awareness about the severity of household air pollution caused by cooking are required at individual levels. Appropriate measures tending to a wide variety of issues related to cooking through awareness, economic development, and renewable energy resources can be extremely beneficial in reducing the possible CV health concerns produced by biomass fuel smoke.

In recent years, China has made significant progress in terms of improving air quality. In 2014, the government proclaimed a battle against air pollution and enacted a national plan to combat it. Since the announcement was made, air pollution levels in the cities of China have dropped by ~32% on average, as per estimates from ground-level sensors. Although these reductions are usually greater than those identified with the satellite-derived pollution information utilized in the AQLI, they would increase life expectancy by 2.3 years if confirmed and sustained over time. India has proclaimed its war against pollution in January 2019, would be on a comparable way if it prevails with regards to meeting its expressed pollution mitigation objective of 20–30%. Pakistan has the potential to encounter similar strides. If Pakistan somehow happened to accomplish a similar 32% pollution reduction experienced in China and to maintain it, its inhabitants would survive 1.2 years longer by and large. This would put the country 52% of the best approach to accomplishing its air quality norm and 43% closer to achieve the WHO recommendation (1, 40).

Our findings have significant ramifications for policymakers and decision-makers in terms of the sustainable environment and health infrastructure. We must realize that the health sector is simply one of many factors to a healthy life expectancy to achieve a health objective with a CRD or CVD focus. Agriculture, environmental, transportation, and economic policies, along with international trade pacts, will influence diet, physical inactivity, environmental sustainability, and access to better health facilities. We must work together and collaborate within regions and disciplines to promote and assert a significant return of interest in respiratory and cardiac health; only then, we will be able to persuade economies and businesses to contribute critical resources to our mutual goals, which is fundamental to population health and wellness in the populace of Pakistan. Eventually, the Government and private organizations in Pakistan should unanimously collaborate, encourage, and concentrate on strategies that can minimize the regional burden of diseases through planned urbanization, sustainable population growth, adoption of clean and renewable energy sources, raising the educational level, enhancing living standards, improving access to quality health services, and investment in public health expenditure to mitigate the risk of air pollution and related mortality.

Besides, researchers have agreed that the MCDA paradigm is productive in environmental healthcare domains and a beneficial decision-making tool since it enables transparency, robustness, and consistency in the context of diverse and contradictory parameters (41, 42). The study outcomes recommend that when confronted with various alternatives of equivalent worth in decision-making situations, researchers should employ MCDA methodologies and tools. This investigation may facilitate researchers with multiple criteria decision-making roadmap to help them enhance the quality of their studies and their understanding of how to use MCDA techniques to evaluate and prioritize the influencing factors of disease mortality in environmental healthcare research. Further, the suggested methodologies provide a valuable tool and additional practical knowledge for policymakers and decision-makers in drawing rational decisions. However, further investigations are necessary to contrast the particularities of MCDA approaches (e.g., preferred inferencing approaches) and enable researchers in selecting the appropriate tool, as there is no rationale for why one MCDA technique is adopted over the other.

## Conclusions

This corroboration adds to the growing body of research demonstrating that exposure to ambient PM_**2.5**_ adversely leads to respiratory and cardiac risks, emphasizing the demand for further air quality improvement in Pakistan. Besides, the suggested methodologies provide a valuable tool and additional practical knowledge for policymakers and decision-makers in drawing rational decisions.

## Data Availability Statement

The data used to support the findings of this study are included within the article.

## Author Contributions

AM, SR, ER, and IH are responsible for conceptualizing the research theme, data collection, and analysis, interpretation of the results, and drafting earlier versions of the manuscript. SR is in charge of project administration and supervision of the overall manuscript. All authors listed have made a substantial, direct, and intellectual contribution to the work and approved it for publication.

## Conflict of Interest

The authors declare that the research was conducted in the absence of any commercial or financial relationships that could be construed as a potential conflict of interest.

## Publisher's Note

All claims expressed in this article are solely those of the authors and do not necessarily represent those of their affiliated organizations, or those of the publisher, the editors and the reviewers. Any product that may be evaluated in this article, or claim that may be made by its manufacturer, is not guaranteed or endorsed by the publisher.
